# Serum Iron Levels and the Risk of Parkinson Disease: A Mendelian Randomization Study

**DOI:** 10.1371/journal.pmed.1001462

**Published:** 2013-06-04

**Authors:** Irene Pichler, Fabiola Del Greco M., Martin Gögele, Christina M. Lill, Lars Bertram, Chuong B. Do, Nicholas Eriksson, Tatiana Foroud, Richard H. Myers, PD GWAS Consortium, Michael Nalls, Margaux F. Keller, International Parkinson's Disease Genomics Consortium, Wellcome Trust Case Control Consortium 2, Beben Benyamin, John B. Whitfield, Genetics of Iron Status Consortium, Peter P. Pramstaller, Andrew A. Hicks, John R. Thompson, Cosetta Minelli

**Affiliations:** 1 Respiratory Epidemiology and Public Health, National Heart and Lung Institute, Imperial College, London, United Kingdom; 2 Neuropsychiatric Genetics Group, Department of Vertebrate Genomics, Max Planck Institute for Molecular Genetics, Berlin, Germany; 3 Department of Neurology, Medical Center of the Johannes Gutenberg-University, Mainz, Germany; 4 23andMe, Inc., Mountain View, California, United States of America; 5 Indiana University School of Medicine, Indianapolis, Indiana, United States of America; 6 Department of Neurology, Boston University School of Medicine, Boston, Massachusetts, United States of America; 7 Laboratory of Neurogenetics, National Institute on Aging, National Institutes of Health, Bethesda, Maryland, United States of America; 8 Department of Biological Anthropology, Temple University, Philadelphia, Pennsylvania, United States of America; 9 Queensland Institute of Medical Research, Brisbane, Queensland, Australia; 10 Queensland Brain Institute, The University of Queensland, Queensland, Australia; 11 Department of Neurology, General Central Hospital, Bolzano, Italy; 12 Department of Neurology, University of Lübeck, Lübeck, Germany; 13 Department of Health Sciences, University of Leicester, Leicester, United Kingdom; Brain and Mind Research Institute, Australia

## Abstract

**Background:**

Although levels of iron are known to be increased in the brains of patients with Parkinson disease (PD), epidemiological evidence on a possible effect of iron blood levels on PD risk is inconclusive, with effects reported in opposite directions. Epidemiological studies suffer from problems of confounding and reverse causation, and mendelian randomization (MR) represents an alternative approach to provide unconfounded estimates of the effects of biomarkers on disease. We performed a MR study where genes known to modify iron levels were used as instruments to estimate the effect of iron on PD risk, based on estimates of the genetic effects on both iron and PD obtained from the largest sample meta-analyzed to date.

**Methods and Findings:**

We used as instrumental variables three genetic variants influencing iron levels, *HFE* rs1800562, *HFE* rs1799945, and *TMPRSS6* rs855791. Estimates of their effect on serum iron were based on a recent genome-wide meta-analysis of 21,567 individuals, while estimates of their effect on PD risk were obtained through meta-analysis of genome-wide and candidate gene studies with 20,809 PD cases and 88,892 controls. Separate MR estimates of the effect of iron on PD were obtained for each variant and pooled by meta-analysis. We investigated heterogeneity across the three estimates as an indication of possible pleiotropy and found no evidence of it. The combined MR estimate showed a statistically significant protective effect of iron, with a relative risk reduction for PD of 3% (95% CI 1%–6%; *p = *0.001) per 10 µg/dl increase in serum iron.

**Conclusions:**

Our study suggests that increased iron levels are causally associated with a decreased risk of developing PD. Further studies are needed to understand the pathophysiological mechanism of action of serum iron on PD risk before recommendations can be made.

*Please see later in the article for the Editors' Summary*

## Abstract 2

In this study, Mendelian randomization was used to study genes known to modify iron levels, and the effect of iron on Parkinson's disease (PD) risk was estimated. Based on estimates of the genetic effects on both iron and PD obtained from the largest sample meta-analyzed to date, the findings suggest that increased iron levels in the blood are associated with a 3% reduction in the risk of Parkinson's disease for every 10 µg/dL increase in iron. The results of this analysis have potentially important implications for future research into the prevention of Parkinson's disease.

*Please see later in the article for the Editors' Summary*

## Editors' Summary

**Background:**

Parkinson disease is a degenerative disorder of the central nervous system caused by the death of dopamine-generating cells in the substania nigra, a region of the midbrain. The earliest symptoms are usually movement-related and include tremor, slow movements, and difficulty walking, and later cognitive and behavioral problems may arise, with dementia commonly occurring in the advanced stages of the disease. Parkinson disease affects around ten million people world-wide and incidence increases with age, with men more affected than women. To date, the causes of Parkinson disease remain unknown although a combination of genetic and environmental factors is thought to play a role. Identifying possible modifiable risks is an important step in the possible prevention of Parkinson disease.

**Why Was This Study Done?:**

Previous studies have shown a possible association between lower blood levels of iron in people with Parkinson disease compared with controls, although the quality of these studies makes this finding difficult to interpret. So in this study, the researchers used a mendelian randomization approach to investigate whether there was any evidence of an effect of blood iron levels on the risk of Parkinson disease and if so to further explore the direction and scale of any link. Mendelian randomization is a method of using measured variation in genes of known function to examine the causal effect of a modifiable exposure on disease in situations where it is inappropriate to perform a randomized controlled trial.

**What Did the Researchers Do and Find?:**

The researchers estimated the effect of blood iron levels on the risk of Parkinson disease using three polymorphisms in two genes, *HFE* and *TMPRSS6*. For each polymorphism, they performed a meta-analysis combining the results of studies investigating the genetic effect on iron levels, which included almost 22,000 people from Europe and Australia, and a meta-analysis of studies investigating the genetic effect on the risk of Parkinson disease, which included a total of 20,809 people with Parkinson disease and 88,892 controls from Europe and North America. They then performed three separate mendelian randomization analyses to estimate the effect of iron on Parkinson disease for the three polymorphisms. By combining the three estimates, they obtained a statistically significant odds ratio of 0.97 for Parkinson disease per 10 µg/dl increase in iron, corresponding to a 3% reduction in the risk of Parkinson disease for every 10 µg/dl increase in blood iron. Since genotype influences on blood iron levels represent differences that generally persist throughout adult life, the combined mendelian randomization estimate reflects an effect of iron over the course of a lifetime.

**What Do These Findings Mean?:**

These findings suggest that increased iron levels in the blood are associated with a 3% reduction in the risk of Parkinson disease for every 10 µg/dl increase in iron. This finding is important as it suggests that increased blood iron levels may have a protective effect against Parkinson disease, although the underlying mechanism remains unclear. Furthermore, although mendelian randomization is an increasingly used approach to address the issue of classical confounding, there may be remaining confounding factors specific of mendelian randomization that may influence the interpretation of this study. Nevertheless, the results of this analysis have potentially important implications for future research into the prevention of Parkinson disease. Further studies on the underlying mechanisms are needed before any specific treatment recommendations can be proposed.

**Additional Information:**

Please access these Web sites via the online version of this summary at http://dx.doi.org/10.1371/journal.pmed.1001462.

- The National Institutes of Neurological Disorder and Stroke, MedlinePlus, and NHS Choices have several pages with comprehensive information on Parkinson disease
- Wikipedia gives an explanation of mendelian randomization (note that Wikipedia is a free online encyclopedia that anyone can edit; available in several languages)

## Introduction

Iron is involved in fundamental biochemical activities, such as oxygen delivery, mitochondrial respiration, and DNA synthesis in almost all cell types. In the brain, iron is a cofactor for a large number of enzymes, including key enzymes of neurotransmitter biosynthesis, such as the tyrosine hydroxylase, which represents the rate-limiting enzyme of dopamine synthesis [1]. However, iron is also potentially toxic as an excess of free iron contributes to the generation of reactive oxygen species and can favor oxidative tissue damage [1]. In the brains of patients with Parkinson disease (PD), increased levels of iron in the substantia nigra (SN) and the lateral globus pallidus have been observed, and yet the mechanisms responsible for this phenomenon are not completely understood [2],[3]. PD is characterized by the rather selective loss of dopaminergic neurons [4] and the presence of α-synuclein-enriched Lewy body inclusions in the SN [5], and several studies have demonstrated that free iron in the SN can enhance the aggregation of α-synuclein and may thus promote the formation of Lewy bodies [1].

Limited epidemiological evidence on the relationship between peripheral blood levels of iron and PD risk is available. A recent meta-analysis of ten studies, with a total of 520 PD cases and 711 controls, showed a trend for lower serum iron levels in PD patients compared with controls, although the difference in iron levels was not statistically significant (standardized mean difference: −0.45; 95% CI −0.98 to 0.08; *p* = 0.09) [6]. However, the very large degree of heterogeneity observed across studies (I^2^: 93%; *p*<0.0001) makes it difficult to interpret these findings.

A major limitation of observational studies is the difficulty in distinguishing between causal and spurious associations due to problems of confounding and reverse causation. Mendelian randomization (MR) is an approach based on the use of genes as instrumental variables, which has been proposed to assess causality and provide estimates of the effect of modifiable intermediate phenotypes on disease unaffected by classical confounding or reverse causation, whenever randomized clinical trials are not feasible [7]. Genes are randomly allocated at conception, so that genetic effects on the intermediate phenotype cannot be affected by classical confounding, such as lifestyle factors, or reverse causation, as in the situation where the phenotype level is influenced by the presence of the disease [8]. For this reason, demonstration that a genetic polymorphism known to modify the phenotype level also modifies the disease risk represents indirect evidence of a causal association between phenotype and disease.

The MR estimate of the effect of the intermediate phenotype on the disease is derived from the estimates of the associations of the polymorphism with both intermediate phenotype and disease. MR, as any other instrumental variable approach, has low statistical power and therefore requires very large sample sizes [9]. The recent availability of large collections of genome-wide data on intermediate phenotypes, such as blood biomarkers, and disease traits within international consortia represents a great opportunity to exploit the potentials of this approach, and indeed MR studies have become increasingly popular over the last few years.

The validity of the MR approach relies on the crucial assumption that the polymorphism acts on the disease only through the intermediate phenotype of interest and not through others (assumption of no pleiotropy) [8]. Evaluating the possibility of pleiotropic effects of the polymorphism is therefore fundamental when using MR, and yet pleiotropy can only be excluded with confidence if the function of the gene and its polymorphisms is completely known, which is rarely the case. This problem can be addressed by using multiple instruments (polymorphisms in multiple genes influencing the same intermediate phenotype), since in the absence of pleiotropy, similar MR estimates should be obtained regardless of the instrument used, so that differences across MR estimates beyond what can be expected by chance can indicate the presence of pleiotropy [10].

In this study, we provide evidence on the presence, direction, and magnitude of a causal effect of serum iron levels on PD risk by performing a MR study, based on iron data in 21,567 individuals from the general population and PD data from 20,809 PD cases and 88,892 controls. We used three polymorphisms as instruments in order both to increase statistical power by combining their MR estimates and to investigate the possible presence of pleiotropy.

## Methods

### Mendelian Randomization Approach

**Figure 1:**
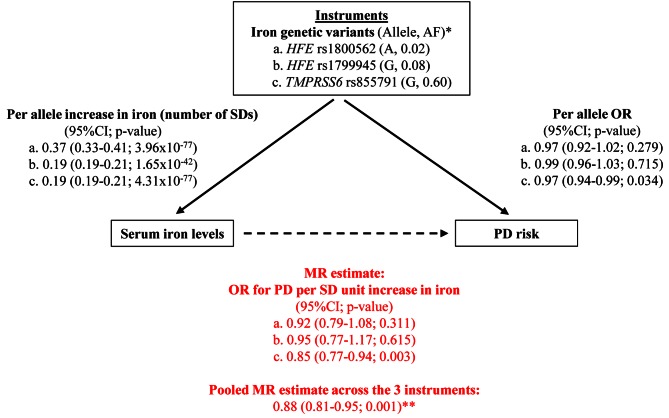
Graphical representation of the MR approach, with all estimates used to derive the final MR estimate. *Reported is the allele that increases iron levels, together with its frequency (AF). **This corresponds approximately to an OR per unit µg/dl increase in iron of 0.997 (95%CI 0.994–0.999), that is 0.3% (0.1%–0.6%) relative reduction in PD risk per 1 µg/dl increase in iron.

The selection of the genes modifying iron levels to be used as instruments in our MR study was based on published results showing that polymorphisms in the hemochromatosis (*HFE*, ENSG00000010704) gene and the transmembrane protease 6 (*TMPRSS6*, ENSG00000187045) gene have the strongest effects on serum iron in the general population of European ancestry [11]. The choice of the polymorphisms within these two genes was based on the findings of a recent large meta-analysis of genome-wide association (GWA) studies on iron levels in the general population (unpublished data). We selected the polymorphisms with the strongest statistical evidence, two for the *HFE* gene, rs1800562 (*C282Y*) and rs1799945 (*H63D*), which are not in linkage disequilibrium (HapMap CEU r^2^<0.01) and therefore represent independent signals of association, and one for the *TMPRSS6* gene, rs855791 (*V736A*) (Figure 1).

Our MR approach was based on the use of aggregate results for both the gene–iron and gene–PD associations: for each polymorphism, we performed a meta-analysis of studies investigating its effect on iron levels and a meta-analysis of studies investigating its effect on PD risk, with no studies contributing to both meta-analyses (see next sections). Three separate MR estimates of the effect of iron on PD were obtained for the three polymorphisms, and they were subsequently pooled by meta-analysis to provide a single MR estimate. Heterogeneity between the three MR estimates was investigated to detect the possible presence of pleiotropy.

### Data on Gene Associations with Iron

Estimates of the effect sizes of the three polymorphisms in *HFE* and *TMPRSS6* on total serum iron levels was based on the findings of a recent GWA meta-analysis on iron parameters performed by the Genetics of Iron Status (GIS) Consortium (Table 1) (unpublished data). The GIS meta-analysis includes ten cohorts from eight participating research groups. The individual datasets included in the meta-analysis are described in Table S1.

**Table 1:** Characteristics of the studies included for the gene–iron and gene–PD associations.

Data Source	*n* studies	Type of Study	Maximum Sample Size
**Gene–iron association**			
GIS Consortium^a^	10	GWA	21,567
**Gene–PD association**			
PDGene database [13]–[21]	9	Candidate gene studies (*HFE* rs1800562 and *HFE* rs1799945)	2,384 cases; 6,908 controls
PD GWAS Consortium [22]	5	GWA	4,238 cases; 4,239 controls
23andMe [23] b	1	GWA	4,127 cases; 62,037 controls
IPDGC [24],[25] c	4	GWA	4,258 cases; 10,152 controls
IPDGC [24],[25]	5	Immunochip genotyping	5,802 cases; 5,556 controls

Details on individual datasets are reported in Text S1 and in Tables S1 and S2.

a Unpublished data. The original sample size was 22,444, but genotype and phenotype data were available only for 21,567.

b 23andMe: slightly expanded version of the cohort used in [23].

c IPDGC: USA-NIA and USA-dbGAP studies were not included in our analysis due to overlap with PD GWAS Consortium; the Icelandic dataset was not available for analysis.

### Data on Gene Associations with PD Risk

To estimate the association of the three polymorphisms with PD risk, we performed a meta-analysis of both candidate gene and GWA studies (Table 1).

Candidate gene studies were identified using PDGene (http://www.pdgene.org), a database providing a regularly updated synopsis of genetic association studies performed in PD [12]. These studies provided data for the two polymorphisms in *HFE*, rs1800562 and rs1799945. A total of nine studies were included in our analysis for both rs1800562 [13]–[20] and rs1799945 [13]–[17],[19]–[21] (Tables 1 and S2).

Three large international GWA studies recently published, the PD GWAS Consortium [22], the 23andMe study [23], and the International Parkinson's Disease Genomics Consortium (IPDGC) [24],[25], provided data for all three polymorphisms (Table 1). The PD GWAS Consortium includes data from five studies: PROGENI/GenePD [26], NIA Phase I [27], NIA Phase II [28], HIHG [29], and NGRC [30]. The 23andMe data come from a slightly expanded version of the cohort used in [23], including more than 4,000 PD cases and 60,000 controls. From the IPDGC, four GWA studies were included in our analysis, together with five studies genotyped with a custom genotyping array (Immunochip Illumina iSelect array); the USA-NIA and the USA-dbGAP studies were not included because of overlap with the PD GWAS dataset, and the Icelandic study was not available for analysis.

A detailed description of the individual datasets is reported in Text S1 and in Table S2.

### Statistical Analyses

GIS meta-analysis results for the gene–iron association were expressed in terms of Z-score, that is the number of standard deviations (SDs) above the mean iron level associated with each copy of the allele.

Study results for the candidate gene studies investigating the gene–PD risk association were obtained either from the PDGene website or directly from the original papers [18]–[20]. For two studies, estimates of the associations of interest were not provided, but they could be calculated from the data reported, by performing a per-genotype analysis based on an additive genetic model [19], or a per-allele analysis when genotype data were not available [20]. For the gene–PD meta-analysis, estimates of the (log) odds ratio (OR) were combined across studies using an inverse-variance-weighted fixed-effect model and assuming an additive genetic model, consistently with the gene–iron meta-analysis.

As for the instrumental variable analysis, an MR estimate of the effect of iron on PD risk was obtained for each of the three instruments separately, and the three estimates were combined using an inverse-variance-weighted fixed-effect meta-analysis. We evaluated the presence and magnitude of heterogeneity across the three instruments with the I^2^ statistics, a measure defined as the percentage of total variation in study estimates explained by heterogeneity rather than sampling error [31]. MR estimates were derived using the Wald-type estimator [32]:
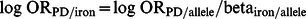
where log OR_PD/iron_ is the (log) increase of PD risk by SD unit increase in iron (MR estimate), log OR_PD/allele_ is the (log) increase in PD risk per allele (gene–PD association), and beta_iron/allele_ is the number of SDs above the mean iron level per allele (gene–iron association). The standard error of the MR estimate was derived using the Delta method [33],[34]. The MR estimate is presented in terms of OR, by exponentiating the log OR_PD/iron_.

We evaluated the strength of each instrument using the F statistics, which is a function of the magnitude and precision of the genetic effect on the biomarker (iron):
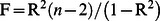
where R^2^ is the variance of iron blood levels explained by the genetic variant and *n* is the sample size for the gene–iron association. We also evaluated the overall F statistics for the three combined instruments assuming that their effects were independent, as are expected to be given that the three gene variants are not in linkage disequilibrium.

A sensitivity analysis was performed to investigate the possible impact on our findings of population stratification in any of the studies included in the gene–iron or gene–PD analyses, by excluding studies which had not adjusted for population stratification.

All analyses were performed using Stata 10 (StataCorp LP).

## Results

### Gene Association with Iron

The GIS meta-analysis for iron levels included 21,567 individuals from Europe and Australia (Table S1). The effect on iron levels, expressed as number of SDs from the mean, was 0.37 (95% CI 0.33–0.41; *p = *4.0×10^−77^) for each copy of the A allele of *HFE* rs1800562, 0.19 (95% CI 0.17–0.21; *p = *1.7×10^−42^) for the G allele of *HFE* rs1799945, and 0.19 (95% CI 0.17–0.21; *p = *4.3×10^−77^) for the G allele of *TMPRSS6* rs855791 (Figure 1; Table S3). With a SD for serum iron levels of 37.6 µg/dl, these figures correspond to an increase in iron per allele of approximately 13.9, 7.1. and 7.1 µg/dl, respectively. *HFE* rs1800562, *HFE* rs1799945, and *TMPRSS6* rs855791 explained 1.7%, 0.9%, and 1.7% of iron total variance, respectively (Table S3).

The F statistics was very high for all genetic variants, as can be expected given the sample size of more than 21,000 individuals [35]: 382, 199, and 379 for *HFE* rs1800562, *HFE* rs1799945, and *TMPRSS6* rs855791, respectively. The F statistics for all combined instruments was 987.

### Gene Association with PD Risk

All datasets available for the analysis of the effects of the three genetic polymorphisms on PD risk (Table S2) were checked for the presence of overlapping studies, and duplicates were removed. The meta-analysis, which included a total of 20,809 PD cases and 88,892 controls from Europe and North America (Table S2), revealed a significant association for *TMPRSS6* rs855791 with PD risk, with an OR of 0.97 (95% CI 0.94–0.99; *p = *0.034) per copy of the G allele. As shown in the Forest plot of the meta-analysis for this polymorphism (Figure S3), there was no statistical evidence of heterogeneity across studies, with a heterogeneity test *p*-value of 0.86 and an I^2^ of 0% (95% CI 0%–85%). In particular, although the 23andMe study was based on self-reported disease status and therefore differed from the rest, its results were consistent with those of the other PD studies. The association with PD risk for the two polymorphisms in *HFE* was not statistically significant, with an OR of 0.97 (95% CI 0.92–1.02; *p = *0.281) for the A allele of rs1800562 and 0.99 (95% CI 0.96–1.03; *p = *0.715) for the G allele of rs1799945 (Figures 1, S1, and S2; Table S4). This might be explained by the much lower statistical power for the two *HFE* variants compared with the *TMPRSS6* variant due to their lower minor allele frequency (1,000 Genomes project: 0.02 and 0.08 versus 0.40), as suggested by their wide confidence intervals.

### Mendelian Randomization Estimate of Iron Association with PD Risk

**Figure 2:**
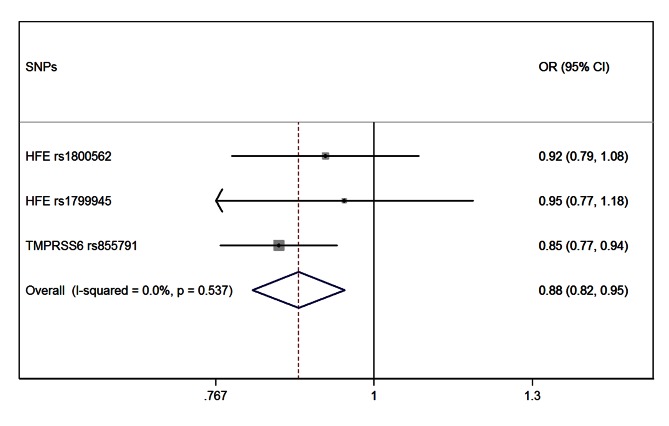
Forest plot of the MR estimates from the three instruments. The size of the squares is proportional to the precision of the MR estimates for each polymorphism, with the horizontal lines indicating their 95% confidence intervals. The combined MR estimate is represented by the centre of the diamond, with the lateral tips indicating its 95% confidence interval. The solid vertical line is the line of no effect.

The meta-analysis of the three MR estimates resulted in a statistically significant combined estimate of 0.88 (95% CI 0.82–0.95; *p = *0.001), representing the OR for PD per SD unit increase in iron (Figure 1). Again, with a SD for iron levels of 37.6 µg/dl, this corresponds approximately to an OR of 0.997 (95% CI 0.994–0.999) per 1 µg/dl increase in iron, that is a 0.3% (95% CI 0.1%–0.6%) relative risk reduction. The Forest plot in Figure 2 shows how the meta-analysis result was driven by the *TMPRSS6* rs855791 variant, and that there was no statistical evidence of heterogeneity across instruments (*p = *0.54; I^2^: 0%, 95% CI 0%–90%), suggesting that the assumption of no pleiotropy might hold.

The sensitivity analysis investigating the impact of population stratification excluded the nine studies from PDGene, which had not reported any adjustment for population stratification, while there were no exclusions from the GIS consortium on iron since all studies had adjusted for population stratification (Table S2). The result of the sensitivity analysis was similar to that of the main analysis, with a combined MR estimate of 0.91 (95% CI 0.83–0.99; *p = *0.032) (Figure S4).

## Discussion

Our study shows a protective effect of serum iron levels on PD, with a 3% (95% CI 1%–6%; *p = *0.001) relative reduction in PD risk per 10 µg/dl increase in iron. If we hypothesise increasing serum iron levels of one SD unit (38 µg/dl in our study) in a population of Caucasians older than 60, where PD risk is around 1% [36], a corresponding relative risk reduction of 12% would translate to a decrease in PD cases from 100/10,000 to 88/10,000. Since genotype influences on serum iron levels represent differences that generally persist throughout adult life, the estimate of our MR study reflects an effect of iron over the course of a lifetime. These findings are important since evidence on the association between serum iron levels and PD risk collected so far has been controversial. Although iron is generally thought of as a risk factor for PD, in line with the well-known phenomenon of iron accumulation in the brain of PD patients [2],[3], epidemiological studies have shown effects of iron in opposite directions. A recent meta-analysis of epidemiological studies suggests a possible protective role of serum iron levels on PD risk, but its findings are difficult to interpret owing to the very large degree of heterogeneity across studies [6]. Epidemiological studies suffer from confounding and reverse causation, which are intrinsic to their observational nature, so that they can hardly provide conclusive evidence on the causality of an observed association. Tobacco smoking and coffee drinking, which have been suggested as protective factors for PD [37],[38], represent two potential confounders for the association between iron and PD, since both might have an effect on iron levels. Nicotine might decrease the availability of free reactive iron [39], and coffee is known to inhibit the intestinal absorption of iron [40],[41]. Reverse causation could also produce spurious associations in epidemiological studies if the phenotype level can be influenced by the presence of the disease. An example is that of monoamine oxidase (MAO) inhibitors used to treat PD. MAO inhibitors may have iron-chelating effects and thus reduce iron blood levels, which could lead to spurious epidemiological evidence of a difference in iron levels between PD cases and controls [42]. Although causality is usually assessed by use of randomized clinical trials, the MR approach represents a valuable alternative whenever these are not feasible [7]. It is based on the concept that genetic variation modifying the concentration of a biomarker should also affect the disease risk if (and only if) the biomarker is directly and causally involved in the disease pathogenesis. Being genes randomly allocated at conception, their effects on biomarkers are unaffected by classical confounding factors and reverse causation [8].

The protective effect of higher serum iron levels on PD risk found in our study may seem somewhat counterintuitive at first sight. However, there are several reports in the literature in line with our findings. A recent study showed a negative correlation between SN echogenicity, a marker for increased SN iron content [43], and serum iron levels in PD patients [44]. A case-control study suggested an increased risk of PD in men who reported multiple recent blood donations and thus experienced depleted systemic iron stores [45], and another study showed an association of anemia experienced early in life with increased PD risk, with the authors hypothesizing that anemia could be a surrogate marker for iron deficiency [46]. Finally, in dietary iron-restricted mice impaired motor behavior and a marked decrease of striatal dopamine levels was observed, which was explained with the fact that iron is essential for the activity of tyrosine hydroxylase, the rate-limiting enzyme in the dopamine synthesis [47]. Consistent with these findings, a recent study performed in Japan found an association between higher iron intake and reduced PD risk [48].

The underlying mechanisms of the protective effect of iron on PD risk observed in our study remains unclear, as does the mechanism that regulates the relationship between serum and brain iron levels. Low peripheral iron levels may reduce the functioning of neuronal enzymes or receptors, since iron is a crucial cofactor of tyrosine hydroxylase [49], plays a role in the synthesis of monoamine neurotransmitters, and is involved in dopaminergic neurodevelopment [50]. Furthermore, low iron levels may decrease neuronal iron storage in the form of ferritin [51], which was found to be inappropriately low in SN neurons in PD [1]. A reduction in ferritin could decrease neuronal iron utilization by decreasing the pool of iron available for neuronal enzymes [47], thus leading to the accumulation of free iron in SN [1]. Similar large-scale MR studies investigating other markers of iron metabolism, such as ferritin and transferrin, could contribute to our understanding of the role of peripheral iron homeostasis in the pathophysiology of PD.

To our knowledge, this is the first MR study aimed at estimating the magnitude of the effect of serum iron levels on PD risk. Previous case-control studies have tried to assess causality and direction of the association by investigating the effect on PD risk of genes involved in iron metabolism and homeostasis, although their findings are somewhat inconsistent with only some supporting the hypothesis of a causal association. Among the many genes evaluated, which include *FTL*, *FTH1*, *TF*, *TFRC*, *IREB2*, *LTF*, *CP*, *FXN*, *HFE* [52], *HPX*, *HAMP*, *HFE2* [53], and *FTMT* [54], only the *G258S* polymorphism in the *TF* gene showed a statistically significant association with PD [17], although the finding was not replicated in a subsequent study [55], and a haplotype in the *SLC11A2* gene was found to occur more frequently in PD [56]. However, all these previous studies were relatively small and therefore underpowered to detect modest genetic effects on PD risk. Our MR study used three polymorphisms in the *HFE* and *TMPRSS6* genes as instruments. Evidence on their association with PD risk was obtained through meta-analysis of several candidate gene studies and three large GWA studies, including a total of more than 20,000 patients and 88,000 controls, which represents the largest PD case-control sample with genetic data meta-analyzed to date. Similarly, estimates of the effect of the three polymorphisms on serum iron levels were based on results from a recent GWA meta-analysis including more than 21,000 individuals. Unlike similar MR investigations that have combined multiple instruments into a single allele score using individual data analyses from all contributing studies, our analyses required only aggregate results for the effect of each genetic variant on both biomarker and disease. This may have practical importance, since it allows inclusion of results from ongoing genetic consortia without requiring further analyses, as well as inclusion of previous findings from published reports. However, methodological work will be needed to assess the relative benefits of the two approaches under different scenarios.

The crucial aspect of a MR study, and more generally of any study based on an instrumental variable approach, is the choice of the gene (instrument) that needs to have a strong effect on the intermediate phenotype of interest. We used three polymorphisms as instrumental variables, since the use of multiple instruments influencing the intermediate phenotype of interest can increase the statistical power of the MR analysis [10]. The instrument strength was high for all of them, as shown by their very large F-statistic values. Two of them, rs1800562 (*C282Y*) and rs1799945 (*H63D*), are non-synonymous polymorphisms in *HFE*, a gene with well known effects in the modulation of iron blood levels [57]. The third non-synonymous polymorphism, rs855791 (*V736A*), is located in *TMPRSS6*, a gene whose role in iron regulation was demonstrated more recently [58]. The two variants in the *HFE* gene are responsible for most cases of hereditary hemochromatosis [59],[60], and they are associated with iron overload when present in the homozygous (*C282Y*/*C282Y*) or compound heterozygous (*C282Y*/*H63D*) state. The *C282Y* variant prevents the altered HFE protein from reaching the cell surface and interacting with the transferrin receptor (TfR) [61],[62]. As a result, iron regulation is disrupted. The exact functional effect of the *H63D* variant is as yet unclear, but some evidence suggests that it may alter an intramolecular salt bridge, possibly affecting the interaction of the HFE protein with the TfR [63]. The *TMPRSS6 V736A* variant was found associated with iron-deficiency anemia [64]. Furthermore, the A allele has been shown to inhibit hepcidin more efficiently than the V allele in in vitro experiments, and to affect hepcidin levels in healthy individuals [65]. Interestingly, *TMPRSS6* rs855791 was by far the most influential and was the one driving the result of the meta-analysis of MR estimates from the three instruments. The wide confidence intervals of the MR estimates for *HFE* rs1800562 and rs1799945 suggest that the power of their MR analysis was very limited due to their low allele frequency. This illustrates the importance of balancing the strength of the effect on the intermediate phenotype with allele frequency and statistical power when choosing the instruments for a MR study.

A potential source of bias specific to MR studies is pleiotropy, whereby the *HFE* or *TMPRSS6* genotypes could influence PD risk through another mechanism that is independent of their effect on serum iron levels. Although we cannot completely exclude pleiotropic effects of the three polymorphisms used in our study because of incomplete knowledge of the underlying biology, we can indirectly investigate the presence of such effects through the simultaneous use of the three polymorphisms as multiple instruments. In a MR study, if all instruments are valid, their MR estimates should differ only as a result of sampling error [10], so that there should be no heterogeneity in the meta-analysis of MR estimates. In our meta-analysis of MR estimates there was no evidence of heterogeneity, although the statistical power to detect heterogeneity is limited when only three estimates are included in the meta-analysis [66]. As more evidence on genes influencing iron blood levels becomes available, MR studies investigating the effects of iron on the risk of PD and other diseases will be able to include many more genetic variants as instruments. This will ensure that pleiotropy can be ruled out with greater confidence. Selection of genes to be used as instruments requires careful consideration, since inclusion of variants with small genetic effects on the biomarker may introduce a “weak instrumental variable” bias [35]. Another potential issue in MR investigations is developmental canalization, the ability to produce the same phenotype regardless of genetic (or environmental) variation. If a genetic polymorphism is expressed during fetal development, compensatory processes may influence development in a way that can protect against the effect of the polymorphism [8]. Although canalization of genetic effects needs to be considered when interpreting MR findings, this problem is very difficult to investigate. Finally, one could speculate that the observed association of the subject's iron-related genotype with PD risk might actually reflect an intrauterine effect of iron due to a similar iron-related maternal genotype. Some evidence suggests that maternal iron deficiency could result in an altered iron status of the newborn, with possible negative effects on the neurophysiologic development [67].

Despite all the possible limitations discussed above, MR offers a valuable approach to derive causal effect estimates whenever randomized trials are very difficult to perform, as in the case of iron and PD. A trial investigating the long-term effect of changes in a subject's iron status, obtained by some means, on the risk of developing PD would require not only a very long follow-up but also a huge sample size, given the low frequency of the disease and the magnitude of the effect that might realistically be expected.

In our study, the MR analysis to combine the OR of the gene–PD association with the effect of the gene–iron association was based on a Wald-type estimator, which works under a “rare disease assumption” that is appropriate in the case of PD. However, the use of a Wald-type estimator for the MR analysis of binary outcomes represents only an approximate method and may produce biased MR estimates [32]. Although such bias has been recently shown to be small, typically within 10% of the MR estimate [68], methods in this area are still under active development.

In summary, our MR study suggests a causal association between increased serum iron levels and decreased risk of developing PD, suggesting that disrupted iron metabolism may be an important factor in the pathogenesis of PD. However, further research is needed to elucidate the pathophysiological mechanism of action underlying our findings. The effect of dietary iron or drugs capable of altering the balance between serum iron and iron storage compartments, might prove to be suitable to test in experimental models. The development of such disease models is therefore necessary before any public health or clinical recommendation can be made for primary prevention in subjects at high risk of developing PD.

## Supporting Information

Figure S1 **Forest plot of the meta-analysis of the studies included for the effect of** ***HFE*** **rs1800562 on PD risk.** The boxes indicate the genetic (additive) effects of individual studies, with the size of the box being inversely proportional to the variance and horizontal lines indicating 95% confidence intervals. The diamond indicates the pooled effect estimate, obtained using inverse-variance weighted fixed-effect meta-analysis, and its 95% confidence interval. The full vertical line shows the value for no effect, as opposed to the dashed line indicating the estimated pooled effect.
(TIF)

Figure S2 **Forest plot of the meta-analysis of the studies included for the effect of** ***HFE*** **rs1799945 on PD risk.** The boxes indicate the genetic (additive) effects of individual studies, with the size of the box being inversely proportional to the variance and horizontal lines indicating 95% confidence intervals. The diamond indicates the pooled effect estimate, obtained using inverse-variance weighted fixed-effect meta-analysis, and its 95% confidence interval. The full vertical line shows the value for no effect, as opposed to the dashed line indicating the estimated pooled effect.
(TIF)

Figure S3 **Forest plot of the meta-analysis of the studies included for the effect of** ***TMPRSS6*** **rs855791 on PD risk.** The boxes indicate the genetic (additive) effects of individual studies, with the size of the box being inversely proportional to the variance and horizontal lines indicating 95% confidence intervals. The diamond indicates the pooled effect estimate, obtained using inverse-variance weighted fixed-effect meta-analysis, and its 95% confidence interval. The full vertical line shows the value for no effect, as opposed to the dashed line indicating the estimated pooled effect.
(TIF)

Figure S4 **Sensitivity analysis: Forest plot of the mendelian randomization estimates after exclusion of nine studies from the PDGene dataset that had not adjusted for population stratification (see Table S2).**
(TIF)

Table S1 **Characteristics and sample size of the individual studies included for the gene–iron association.** In all studies, the analyses were adjusted for age and sex, as well as for the first five MDS (multidimensional scaling) or principal components to control for population stratification.
(DOC)

Table S2 **Characteristics and sample size of the individual studies included for the gene–PD association.**
(DOC)

Table S3 **Gene–iron association: GIS-consortium meta-analysis.** The effect size for the genetic effects on iron levels is expressed as number of SDs from the mean (Z-scores).
(DOC)

Table S4 **Gene–PD association: meta-analysis of all available candidate gene and GWA studies.**
(DOC)

Text S1 **Detailed description of the studies included in the three GWA investigations of PD risk.**
(DOC)

## Abbreviations

GIS: Genetics of Iron Status Consortium
GWA: genome-wide association
IPDGC: International Parkinson's Disease Genomics Consortium
MR: mendelian randomization
PD: Parkinson disease
SD: standard deviation
SN: substantia nigra
