# Correction: Serum Iron Levels and the Risk of Parkinson Disease: A Mendelian Randomization Study

**DOI:** 10.1371/annotation/c4d81646-0c0e-4a3e-9425-b220bae2d8b6

**Published:** 2013-06-10

**Authors:** Irene Pichler, Fabiola Del Greco M., Martin Gögele, Christina M. Lill, Lars Bertram, Chuong B. Do, Nicholas Eriksson, Tatiana Foroud, Richard H. Myers, PD GWAS Consortium, Michael Nalls, Margaux F. Keller, International Parkinson's Disease Genomics Consortium, Wellcome Trust Case Control Consortium 2, Beben Benyamin, John B. Whitfield, Genetics of Iron Status Consortium, Peter P. Pramstaller, Andrew A. Hicks, John R. Thompson, Cosetta Minelli

The affiliation currently reads:

Irene Pichler, Fabiola Del Greco M., Martin Gögele, Peter P. Pramstaller, Andrew A. Hicks, Cosetta Minelli: 

Respiratory Epidemiology and Public Health, National Heart and Lung Institute, Imperial College, London, United Kingdom

The affiliation should read:

Irene Pichler, Fabiola Del Greco M., Martin Gögele, Peter P. Pramstaller, Andrew A. Hicks, Cosetta Minelli:

Center for Biomedicine, European Academy Bozen/Bolzano (EURAC), Bolzano, Italy (affiliated institute of the University of Lubeck, Lubeck, Germany).

Cosetta Minelli:

Respiratory Epidemiology and Public Health, National Heart and Lung Institute, Imperial College, London, United Kingdom.

