# Supplementary figures and images for: Serum Iron Levels and the Risk of Parkinson Disease: A Mendelian Randomization Study
Source: PLoS Med. 2013 Jun 4;10(6):e1001462. doi: 10.1371/journal.pmed.1001462 (PMC3672214; doi:10.1371/journal.pmed.1001462)

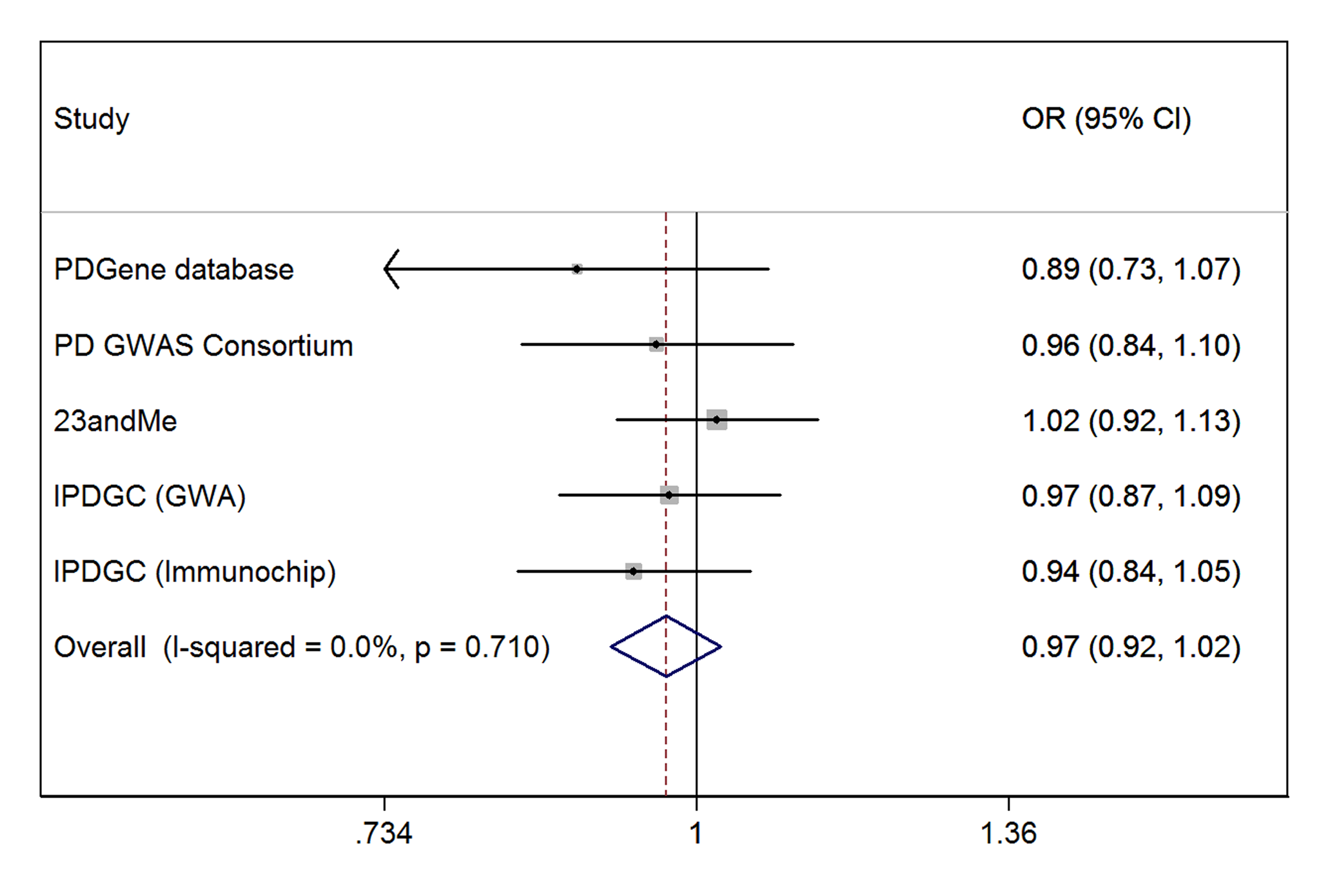

Supplement: Figure S1 — Forest plot of the meta-analysis of the studies included for the effect of HFE rs1800562 on PD risk. The boxes indicate the genetic (additive) effects of individual studies, with the size of the box being inversely proportional to the variance and horizontal lines indicating 95% confidence intervals. The diamond indicates the pooled effect estimate, obtained using inverse-variance weighted fixed-effect meta-analysis, and its 95% confidence interval. The full vertical line shows the value for no effect, as opposed to the dashed line indicating the estimated pooled effect. (TIF) [file pmed.1001462.s001.tif]

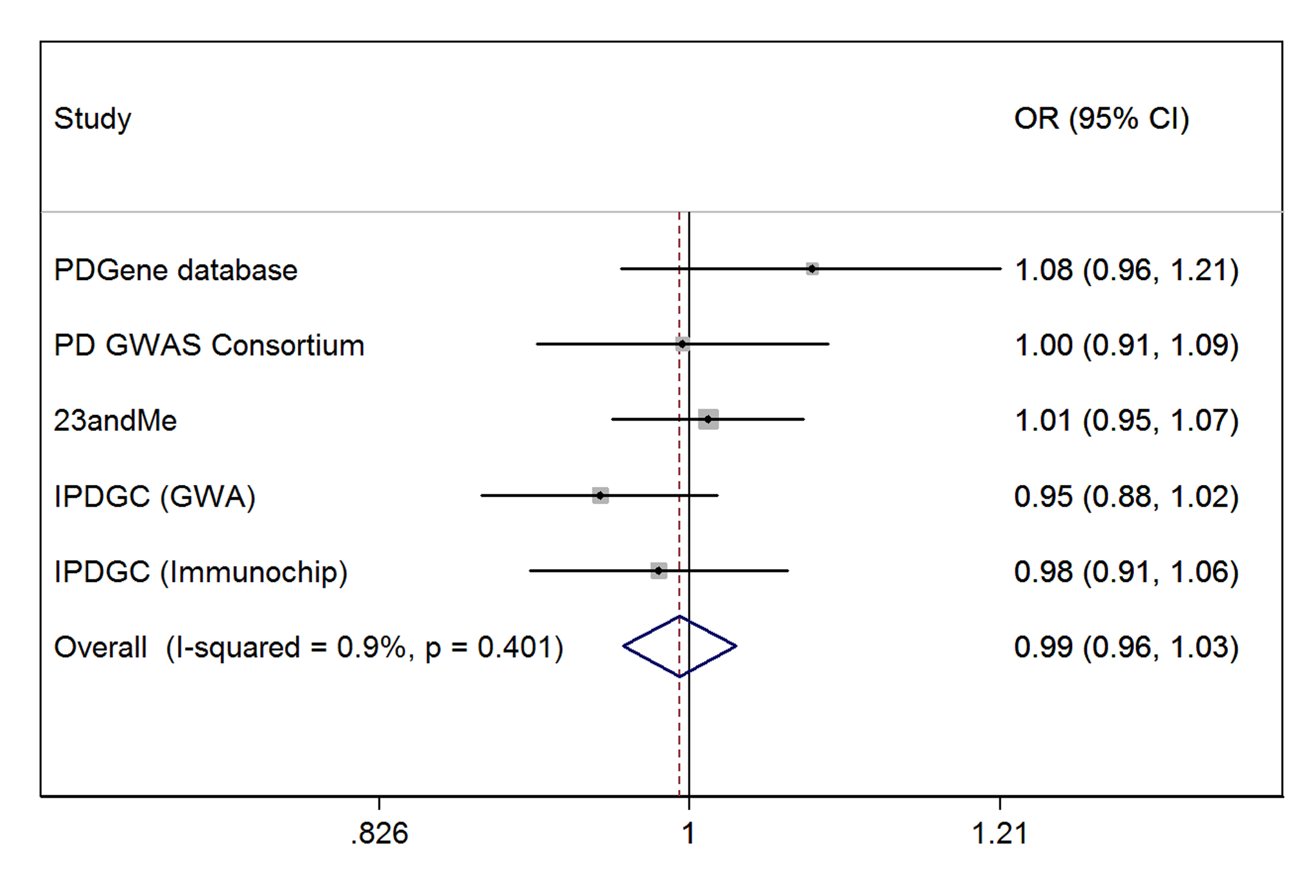

Supplement: Figure S2 — Forest plot of the meta-analysis of the studies included for the effect of HFE rs1799945 on PD risk. The boxes indicate the genetic (additive) effects of individual studies, with the size of the box being inversely proportional to the variance and horizontal lines indicating 95% confidence intervals. The diamond indicates the pooled effect estimate, obtained using inverse-variance weighted fixed-effect meta-analysis, and its 95% confidence interval. The full vertical line shows the value for no effect, as opposed to the dashed line indicating the estimated pooled effect. (TIF) [file pmed.1001462.s002.tif]

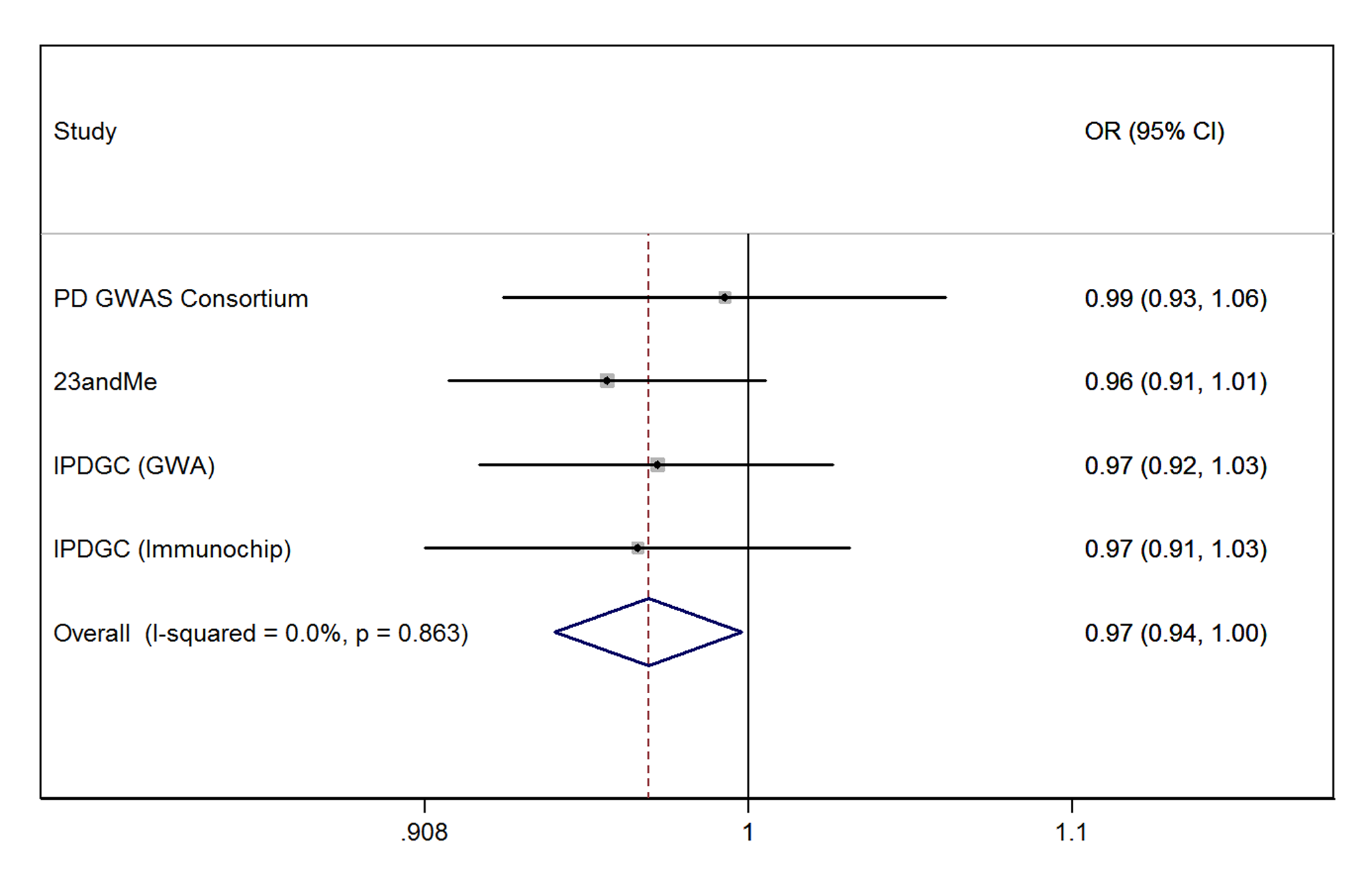

Supplement: Figure S3 — Forest plot of the meta-analysis of the studies included for the effect of TMPRSS6 rs855791 on PD risk. The boxes indicate the genetic (additive) effects of individual studies, with the size of the box being inversely proportional to the variance and horizontal lines indicating 95% confidence intervals. The diamond indicates the pooled effect estimate, obtained using inverse-variance weighted fixed-effect meta-analysis, and its 95% confidence interval. The full vertical line shows the value for no effect, as opposed to the dashed line indicating the estimated pooled effect. (TIF) [file pmed.1001462.s003.tif]

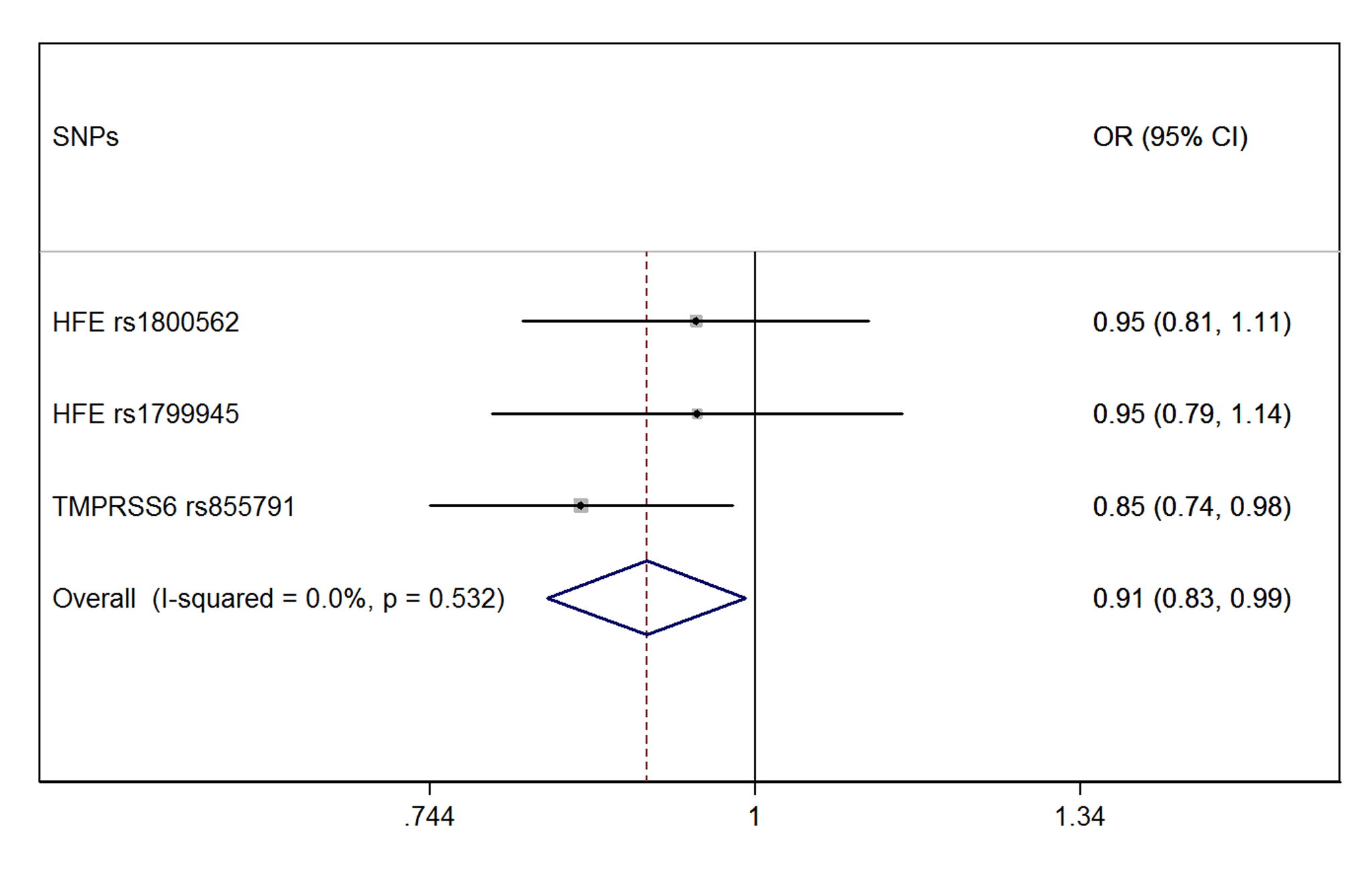

Supplement: Figure S4 — Sensitivity analysis: Forest plot of the mendelian randomization estimates after exclusion of nine studies from the PDGene dataset that had not adjusted for population stratification (see Table S2). (TIF) [file pmed.1001462.s004.tif]
