# Supplementary material for: Serum Iron Levels and the Risk of Parkinson Disease: A Mendelian Randomization Study
Source: PLoS Med. 2013 Jun 4;10(6):e1001462. doi: 10.1371/journal.pmed.1001462 (PMC3672214; doi:10.1371/journal.pmed.1001462)
Supplement: Table S1 — Characteristics and sample size of the individual studies included for the gene–iron association. In all studies, the analyses were adjusted for age and sex, as well as for the first five MDS (multidimensional scaling) or principal components to control for population stratification. (DOC) [file pmed.1001462.s005.doc]

**Table S1.** Characteristics and sample size of the individual studies included for the gene-iron association. The analyses were adjusted for age and sex, as well as for the first five MDS (multidimensional scaling) or principal components to control for population stratification.

| **Data source** | **N. studies** | **Country** | **Type of study** | **Study design** | **Sample size^1^** |
| --- | --- | --- | --- | --- | --- |
| **Genetics of Iron Status (GIS) Consortium^2^** | 10 |  | GWA | M-A of two family-based and eight population-based studies | 22,444 |
| Australia-Adult | 1 | Australia | GWA | Family based study | 9,148 |
| Australia-Adolescent | 1 | Australia | GWA | Family based study | 2,544 |
| Estonia | 1 | Estonia | GWA | Population-based study | 893 |
| KORA | 1 | Germany | GWA | Population-based study | 1,809 |
| Milano | 1 | Italy | GWA | Population-based study | 1,659 |
| Nijmegen | 1 | The Netherlands | GWA | Population-based study | 1,791 |
| MICROS | 1 | Italy | GWA | Population-based study | 1,218 |
| ERF/Rotterdam | 1 | The Netherlands | GWA | Population-based study | 871 |
| KORA F3 | 1 | Germany | GWA | Population-based study | 1,634 |
| BHS-WA | 1 | Australia | GWA | Population-based study | 877 |

^1^ The original sample size was 22,444, but genotype and phenotype data were available only for 21,567 (see Table S3).

^2^ Personal communication B. Benyamin.
