# Supplementary material for: Serum Iron Levels and the Risk of Parkinson Disease: A Mendelian Randomization Study
Source: PLoS Med. 2013 Jun 4;10(6):e1001462. doi: 10.1371/journal.pmed.1001462 (PMC3672214; doi:10.1371/journal.pmed.1001462)
Supplement: Table S2 — Characteristics and sample size of the individual studies included for the gene–PD association. (DOC) [file pmed.1001462.s006.doc]

**Table S2.** Characteristics and sample size of the individual studies included for the gene-PD association.

| **Data source** | **N. studies** | **Country** | **Type of study** | **Study design** | **Sample size** | **Covariates** |
| --- | --- | --- | --- | --- | --- | --- |
| **PDGene database** | 9 |  | candidate gene studies | M-A | 2,384 cases; 6,908 controls | no covariates |
| Greco V et al. 2011 [12] |  | Italy | candidate gene study (*HFE* rs1800562 and *HFE* rs1799945) | case / control | 181 cases; 180 controls | no covariates |
| Halling J et al. 2008 [13] |  | Faroe Islands | candidate gene study (*HFE* rs1800562 and *HFE* rs1799945) | case / control | 79 cases; 154 controls | no covariates |
| Guerreiro RJ et al. 2006 [14] |  | Portugal | candidate gene study (*HFE* rs1800562 and *HFE* rs1799945) | case / control | 132 cases; 115 controls | no covariates |
| Dekker MC et al. 2003 [15] |  | The Netherlands | candidate gene study (*HFE* rs1800562 and *HFE* rs1799945) | case / control | 197 cases; 2,914 controls | no covariates |
| Borie C et al. 2002 [16] |  | France | candidate gene study (*HFE* rs1800562 and *HFE* rs1799945) | case / control | 216 cases; 193 controls | no covariates |
| Aamodt AH et al. 2007 [17] |  | Norway | candidate gene study (*HFE* rs1800562 and *HFE* rs1799945) | case / control | 388 cases; 505 controls | no covariates |
| Biasiotto G et al. 2008 [18] |  | Italy | candidate gene study (*HFE* rs1800562 and *HFE* rs1799945) | case / control | 475 cases; 2,100 controls | no covariates |
| Buchanan DD et al. 2002 [19] |  | Australia | candidate gene study (*HFE* rs1800562) | case / control | 438 cases; 485 controls | no covariates |
| Akbas N et al. 2006 [20] |  | Germany | candidate gene study (*HFE* rs1799945) | case / control | 278 cases; 262 controls | no covariates |
| **PD GWAS Consortium** [6] | 5 |  | GWA | M-A | 4,238 cases; 4,239 controls | sex, (age), PC |
| PROGENI/GenePD [3] |  | USA, Germany, Italy, UK, Canada, Australia | GWA | case / control | 840 cases; 862 controls | sex, age, PC |
| NIA Phase I [1] |  | USA | GWA | case / control | 245 cases; 256 controls | sex, PC |
| NIA Phase II [2] |  | USA | GWA | case / control | 618 cases; 520 controls | sex, age, PC |
| HIHG [4] |  | USA | GWA | case / control | 579 cases; 619 controls | sex, PC |
| NGRC [5] |  | USA | GWA | case / control | 1,956 cases; 1,982 controls | sex, PC |
| **23andMe^1^** [9] | 1 | Europe, USA | GWA | case / control | 4,127 cases; 62,037 controls | sex, age, five PC |
| **IPDGC^2^** [10,11] | 9 |  | GWA / Immunochip genotyping | M-A | 10,060 cases; 15,708 controls | two PC |
| United Kingdom (stage I) |  | United Kingdom | GWA | case / control | 1,705 cases; 5,200 controls | two PC |
| German (stage I) |  | Germany | GWA | case / control | 742 cases; 944 controls | two PC |
| French (stage I) |  | France | GWA | case / control | 1,039 cases; 1,984 controls | two PC |
| Dutch (stage II) |  | The Netherlands | GWA | case / control | 772 cases; 2,024 controls | two PC |
| USA (stage II) |  | USA | Immunochip genotyping | case / control | 2,807 cases; 2,215 controls | two PC |
| United Kingdom (stage II) |  | United Kingdom | Immunochip genotyping | case / control | 1,271 cases; 1,864 controls | two PC |
| Dutch (stage II) |  | The Netherlands | Immunochip genotyping | case / control | 304 cases; 402 controls | two PC |
| French (stage II) |  | France | Immunochip genotyping | case / control | 267 cases; 363 controls | two PC |
| German (stage II) |  | Germany | Immunochip genotyping | case / control | 1,153 cases; 712 controls | two PC |

PC: principal components

^1^ 23andMe: slightly expanded version of the cohort used in [9].

^2^ IPDGC (International Parkinson’s Disease Genomics Consortium): USA-NIA and USA-dbGAP studies were not included in our analysis due to overlap with PD GWAS Consortium; the Icelandic dataset was not available for analysis.
