# Supplementary material for: Serum Iron Levels and the Risk of Parkinson Disease: A Mendelian Randomization Study
Source: PLoS Med. 2013 Jun 4;10(6):e1001462. doi: 10.1371/journal.pmed.1001462 (PMC3672214; doi:10.1371/journal.pmed.1001462)
Supplement: Table S3 — Gene–iron association: GIS-consortium meta-analysis. The effect size for the genetic effects on iron levels is expressed as number of SDs from the mean (Z-scores). (DOC) [file pmed.1001462.s007.doc]

**Table S3.** Gene-iron association in the dataset of the GIS-consortium. The effect size for the genetic effects on iron levels is expressed as number of SDs from the mean (Z-scores).

| **SNP** | **Chr.** | **Gene** | **Ref. allele / other** | **Frequency ref. allele** | **Tot. sample size** | **Beta (95%CI)** | **p-value** | **% Var.** |
| --- | --- | --- | --- | --- | --- | --- | --- | --- |
| rs1800562 | 6 | *HFE* | A / G | 0.02 | 21,567 | 0.37 (0.33-0.41) | 3.96x10^-77^ | 1.739 |
| rs1799945 | 6 | *HFE* | G / C | 0.08 | 21,567 | 0.19 (0.17-0.21) | 1.65x10^-42^ | 0.915 |
| rs855791 | 22 | *TMPRSS6* | G / A | 0.6 | 21,567 | 0.19 (0.17-0.21) | 4.31x10^-77^ | 1.724 |

Chr., chromosome; SE, standard error; ref. allele, reference allele.

% Var., percentage variance explained.

Frequency ref. allele from 1000 Genomes project.
