# Supplementary material for: Serum Iron Levels and the Risk of Parkinson Disease: A Mendelian Randomization Study
Source: PLoS Med. 2013 Jun 4;10(6):e1001462. doi: 10.1371/journal.pmed.1001462 (PMC3672214; doi:10.1371/journal.pmed.1001462)
Supplement: Table S4 — Gene–PD association: meta-analysis of all available candidate gene and GWA studies. (DOC) [file pmed.1001462.s008.doc]

**Table S4.** Gene-PD association: meta-analysis of all available candidate gene and GWA studies.

| **SNP** | **Chr.** | **Gene** | **Ref. allele / other** | **Frequency ref. allele** | **Tot. cases/ tot. controls** | **OR (95%CI)** | **p-value** |
| --- | --- | --- | --- | --- | --- | --- | --- |
| rs1800562 | 6 | *HFE* | A / G | 0.02 | 20,531 / 88,630 | 0.97 (0.92-1.02) | 0.281 |
| rs1799945 | 6 | *HFE* | G / C | 0.08 | 20,371 / 88,407 | 0.99 (0.96-1.03) | 0.715 |
| rs855791 | 22 | *TMPRSS6* | G / A | 0.6 | 18,425 / 81,984 | 0.97 (0.94-1.00) | 0.034 |

Chr., chromosome; SE, standard error; ref. allele, reference allele.

Frequency ref. allele from 1000 Genomes project.
