# Supplementary material for: Serum Iron Levels and the Risk of Parkinson Disease: A Mendelian Randomization Study
Source: PLoS Med. 2013 Jun 4;10(6):e1001462. doi: 10.1371/journal.pmed.1001462 (PMC3672214; doi:10.1371/journal.pmed.1001462)
Supplement: Text S1 — Detailed description of the studies included in the three GWA investigations of PD risk. (DOC) [file pmed.1001462.s009.doc]

# SUPPORTING INFORMATION

**PD GWAS Consortium**

For this dataset (4,238 cases and 4,239 controls), two publicly available [1,2] and three additional GWA studies [3-5] were meta-analyzed [6]. All studies employed standard UK Brain Bank criteria [7] for the diagnosis of PD, with a modification to allow cases with a family history of PD to be included. PD cases with a reported age of onset below 18 years of age were removed (n=17). When data were available, any PD cases known to carry a causative mutation, either two *Parkin* mutations or a single *LRRK2* mutation, were excluded from the analysis (n=57).

*PROGENI/GenePD* [3]

PD cases were selected from the PROGENI and GenePD studies of familial PD. Both studies ascertained multiplex PD families consisting of at least a sibling pair, both of whom were reported to be affected with PD. Control samples were obtained from the NINDS Human Genetics Resource Center at the Coriell Institute, Coriell Cell Repositories (Camden, NJ).

*NIA Phase I* [1]

PD samples were derived from the NINDS Neurogenetics repository hosted by the [Coriell Institute for Medical research](http://www.sciencedirect.com/science?_ob=RedirectURL&_method=externObjLink&_locator=url&_issn=14744422&_origin=article&_zone=art_page&_plusSign=%2B&_targetURL=http%253A%252F%252Fccr.coriell.org) (NJ, USA). For the PD cohort, blood was obtained from unique and unrelated white individuals with idiopathic PD. Both those with and without a reported family history of PD were included. For the control population, blood samples were drawn from neurologically normal, unrelated, white individuals at many different sites within the USA.

*NIA Phase II* [2]

PD patients were derived from the NINDS Neurogenetics Repository at the Coriell Institute for Medical research (NJ, USA). In addition, 75 PD cases collected by a movement disorders specialist in the Laboratory of Neurogenetics were included. All patients were Caucasian individuals with idiopathic PD from the USA.

*MIHG* [4]

Samples in the MIHG GWAS include individuals with PD collected by one of 13 ascertainment centers in the PD Genetics Collaboration [8] or by the Morris K. Udall Parkinson Disease Center of Excellence ascertainment core. These participants were recruited by participating movement disorder and neurology clinics, referrals, and advertisements. Unaffected spouse and friend controls were recruited when available and willing to participate.

*NGRC* [5]

PD patients and control subjects were recruited from eight NGRC-affiliated neurology clinics in Oregon, Washington, Georgia and New York. Controls were community volunteers and patient spouses.

**23andMe**

PD patients of the 23andMe study were recruited through a targeted email campaign together with the Michael J. Fox Foundation, the Parkinson’s Institute and Clinical Center, and many other PD patient groups and clinics [9]. Patients who stated in an online screening questionnaire that they had been diagnosed with PD were offered the 23andMe Personal Genome Service. Controls were drawn from the customer database of the 23andMe company. Individuals included in the PD GWA study were selected for being of primarily European ancestry, and overlapping samples with publically available PD studies from dbGAP were removed. The dataset available for our study included 4,127 cases and 62,037 controls from this growing study.

**International Parkinson’s Disease Genomics Consortium, IPDGC**

From IPDGC [10,11], we included four GWA studies (United Kingdom, German, French, and Dutch datasets) with a total of 4,258 cases and 10,152 controls in our meta-analysis. In addition, five studies genotyped with a custom genotyping array (Immunochip Illumina iSelect array) with genotypes for all three genetic variants were available for our study. These studies consisted of a total of 5,802 cases and 5,556 control samples from USA, United Kingdom, Netherlands, Germany, and France. Details of the studies included are reported below and summarized in Table S2.

*United Kingdom (UK) dataset*

For the UK dataset sample recruitment mostly targeted sporadic cases without familial history of PD. Half of the case collection was tested for the highly penetrant G0219S variant in the *LRRK2* gene and carriers were excluded from the GWA scan. The control set is a shared resource of UK samples (1958 British Birth Cohort and blood donors recruited by the National Blood Services) genotyped by the Wellcome Trust Case Control Consortium.

*German dataset*

The German dataset was collected by movement disorder specialists of the Universities of Munich and Tübingen in Southern Germany. The control dataset was derived from the population based studies KORA and Popgen.

*French dataset*

The patients for the French dataset were recruited through the French network for the study of Parkinson’s disease Genetics (GPD). The patients were enriched for cases with a positive family history of PD. The controls were derived from the French Three-City (3C) cohort, a population-based, prospective study of relationship between vascular factors and dementia.

*Dutch dataset*

The PD patients were recruited from four different centers within the Netherlands (Scales for Outcomes in Parkinson's disease, SCOPA, [http://www.scopa-propark.eu](http://www.scopa-propark.eu/); the Academic Medical Center Amsterdam, AMC, [http://www.amc.uva.nl](http://www.amc.uva.nl/); the Parkinson Centrum Nijmegen, ParC, [http://www.umcn.nl](http://www.umcn.nl/); and the VU University medical centre, VUmc, [http://www.vumc.nl](http://www.vumc.nl/)). Genotyping data from control participants from the Rotterdam study III (ERGO Young) were used as control population.

*Studies genotyped with Immunochip*

The US dataset consisted of samples collected in the Parkinson’s, Genes and Environment (PAGE) and PostCept Studies, as well as additional samples from the Washington University of Saint Louis and the Coriell Repository. The UK dataset consisted of samples contributed by the University College London, Cardiff University and Wellcome Trust population control samples. In addition, Dutch, German, and French case-control samples were available.

**Table S1.** Characteristics and sample size of the individual studies included for the gene-iron association. In all studies the analyses were adjusted for age and sex, as well as for the first five MDS (multidimensional scaling) or principal components to control for population stratification.

| **Data source** | **N. studies** | **Country** | **Type of study** | **Study design** | **Sample size^1^** |
| --- | --- | --- | --- | --- | --- |
| **Genetics of Iron Status (GIS) Consortium^2^** | 10 |  | GWA | M-A of two family-based and eight population-based studies | 22,444 |
| Australia-Adult |  | Australia | GWA | Family based study | 9,148 |
| Australia-Adolescent |  | Australia | GWA | Family based study | 2,544 |
| Estonia |  | Estonia | GWA | Population-based study | 893 |
| KORA |  | Germany | GWA | Population-based study | 1,809 |
| Milano |  | Italy | GWA | Population-based study | 1,659 |
| Nijmegen |  | The Netherlands | GWA | Population-based study | 1,791 |
| MICROS |  | Italy | GWA | Population-based study | 1,218 |
| ERF/Rotterdam |  | The Netherlands | GWA | Population-based study | 871 |
| KORA F3 |  | Germany | GWA | Population-based study | 1,634 |
| BHS-WA |  | Australia | GWA | Population-based study | 877 |

^1^ The original sample size was 22,444, but genotype and phenotype data were available only for 21,567 (see Table S3).

^2^ Unpublished data.

**Table S2.** Characteristics and sample size of the individual studies included for the gene-PD association.

| **Data source** | **N. studies** | **Country** | **Type of study** | **Study design** | **Sample size** | **Covariates** |
| --- | --- | --- | --- | --- | --- | --- |
| **PDGene database** | 9 |  | candidate gene studies | M-A | 2,384 cases; 6,908 controls | / |
| Greco V et al. 2011 [12] |  | Italy | candidate gene study (*HFE* rs1800562 and *HFE* rs1799945) | case / control | 181 cases; 180 controls | / |
| Halling J et al. 2008 [13] |  | Faroe Islands | candidate gene study (*HFE* rs1800562 and *HFE* rs1799945) | case / control | 79 cases; 154 controls | / |
| Guerreiro RJ et al. 2006 [14] |  | Portugal | candidate gene study (*HFE* rs1800562 and *HFE* rs1799945) | case / control | 132 cases; 115 controls | / |
| Dekker MC et al. 2003 [15] |  | The Netherlands | candidate gene study (*HFE* rs1800562 and *HFE* rs1799945) | case / control | 197 cases; 2,914 controls | / |
| Borie C et al. 2002 [16] |  | France | candidate gene study (*HFE* rs1800562 and *HFE* rs1799945) | case / control | 216 cases; 193 controls | / |
| Aamodt AH et al. 2007 [17] |  | Norway | candidate gene study (*HFE* rs1800562 and *HFE* rs1799945) | case / control | 388 cases; 505 controls | / |
| Biasiotto G et al. 2008 [18] |  | Italy | candidate gene study (*HFE* rs1800562 and *HFE* rs1799945) | case / control | 475 cases; 2,100 controls | / |
| Buchanan DD et al. 2002 [19] |  | Australia | candidate gene study (*HFE* rs1800562) | case / control | 438 cases; 485 controls | / |
| Akbas N et al. 2006 [20] |  | Germany | candidate gene study (*HFE* rs1799945) | case / control | 278 cases; 262 controls | / |
| **PD GWAS Consortium** [6] | 5 |  | GWA | M-A | 4,238 cases; 4,239 controls | sex, (age), PC |
| PROGENI/GenePD [3] |  | USA, Germany, Italy, UK, Canada, Australia | GWA | case / control | 840 cases; 862 controls | sex, age, PC |
| NIA Phase I [1] |  | USA | GWA | case / control | 245 cases; 256 controls | sex, PC |
| NIA Phase II [2] |  | USA | GWA | case / control | 618 cases; 520 controls | sex, age, PC |
| HIHG [4] |  | USA | GWA | case / control | 579 cases; 619 controls | sex, PC |
| NGRC [5] |  | USA | GWA | case / control | 1,956 cases; 1,982 controls | sex, PC |
| **23andMe^1^** [9] | 1 | Europe, USA | GWA | case / control | 4,127 cases; 62,037 controls | sex, age, five PC |
| **IPDGC^2^** [10,11] | 9 |  | GWA / Immunochip genotyping | M-A | 10,060 cases; 15,708 controls | two PC |
| United Kingdom (stage I) |  | United Kingdom | GWA | case / control | 1,705 cases; 5,200 controls | two PC |
| German (stage I) |  | Germany | GWA | case / control | 742 cases; 944 controls | two PC |
| French (stage I) |  | France | GWA | case / control | 1,039 cases; 1,984 controls | two PC |
| Dutch (stage II) |  | The Netherlands | GWA | case / control | 772 cases; 2,024 controls | two PC |
| USA (stage II) |  | USA | Immunochip genotyping | case / control | 2,807 cases; 2,215 controls | two PC |
| United Kingdom (stage II) |  | United Kingdom | Immunochip genotyping | case / control | 1,271 cases; 1,864 controls | two PC |
| Dutch (stage II) |  | The Netherlands | Immunochip genotyping | case / control | 304 cases; 402 controls | two PC |
| French (stage II) |  | France | Immunochip genotyping | case / control | 267 cases; 363 controls | two PC |
| German (stage II) |  | Germany | Immunochip genotyping | case / control | 1,153 cases; 712 controls | two PC |

PC: principal components

^1^ 23andMe: slightly expanded version of the cohort used in [9].

^2^ IPDGC (International Parkinson’s Disease Genomics Consortium): USA-NIA and USA-dbGAP studies were not included in our analysis due to overlap with PD GWAS Consortium; the Icelandic dataset was not available for analysis.

**Table S3.** Gene-iron association: GIS-consortium meta-analysis. The effect size for the genetic effects on iron levels is expressed as number of SDs from the mean (Z-scores).

| **SNP** | **Chr.** | **Gene** | **Ref. allele / other** | **Frequency ref. allele** | **Tot. sample size** | **Beta (95%CI)** | **p-value** | **% Var.** |
| --- | --- | --- | --- | --- | --- | --- | --- | --- |
| rs1800562 | 6 | *HFE* | A / G | 0.02 | 21,567 | 0.37 (0.33-0.41) | 3.96x10^-77^ | 1.739 |
| rs1799945 | 6 | *HFE* | G / C | 0.08 | 21,567 | 0.19 (0.17-0.21) | 1.65x10^-42^ | 0.915 |
| rs855791 | 22 | *TMPRSS6* | G / A | 0.6 | 21,567 | 0.19 (0.17-0.21) | 4.31x10^-77^ | 1.724 |

Chr., chromosome; SE, standard error; ref. allele, reference allele.

% Var., percentage variance explained.

Frequency ref. allele from 1000 Genomes project.

**Table S4.** Gene-PD association: meta-analysis of all available candidate gene and GWA studies.

| **SNP** | **Chr.** | **Gene** | **Ref. allele / other** | **Frequency ref. allele** | **Tot. cases/ tot. controls** | **OR (95%CI)** | **p-value** |
| --- | --- | --- | --- | --- | --- | --- | --- |
| rs1800562 | 6 | *HFE* | A / G | 0.02 | 20,531 / 88,630 | 0.97 (0.92-1.02) | 0.281 |
| rs1799945 | 6 | *HFE* | G / C | 0.08 | 20,371 / 88,407 | 0.99 (0.96-1.03) | 0.715 |
| rs855791 | 22 | *TMPRSS6* | G / A | 0.6 | 18,425 / 81,984 | 0.97 (0.94-1.00) | 0.034 |

Chr., chromosome; SE, standard error; ref. allele, reference allele.

Frequency ref. allele from 1000 Genomes project.

**Figure S1.** Forest plot of the meta-analysis of the studies included for the effect of *HFE* rs1800562 on PD risk. The boxes indicate the genetic (additive) effects of individual studies, with the size of the box being inversely proportional to the variance and horizontal lines indicating 95% confidence intervals. The diamond indicates the pooled effect estimate, obtained using inverse-variance weighted fixed-effect meta-analysis, and its 95% confidence interval. The full vertical line shows the value for no effect, as opposed to the dashed line indicating the estimated pooled effect.

**Figure S2.** Forest plot of the meta-analysis of the studies included for the effect of *HFE* rs1799945 on PD risk. The boxes indicate the genetic (additive) effects of individual studies, with the size of the box being inversely proportional to the variance and horizontal lines indicating 95% confidence intervals. The diamond indicates the pooled effect estimate, obtained using inverse-variance weighted fixed-effect meta-analysis, and its 95% confidence interval. The full vertical line shows the value for no effect, as opposed to the dashed line indicating the estimated pooled effect.

**Figure S3.** Forest plot of the meta-analysis of the studies included for the effect of *TMPRSS6* rs855791 on PD risk. The boxes indicate the genetic (additive) effects of individual studies, with the size of the box being inversely proportional to the variance and horizontal lines indicating 95% confidence intervals. The diamond indicates the pooled effect estimate, obtained using inverse-variance weighted fixed-effect meta-analysis, and its 95% confidence interval. The full vertical line shows the value for no effect, as opposed to the dashed line indicating the estimated pooled effect.

**Figure S4.** Sensitivity analysis: Forest plot of the mendelian randomization estimates after exclusion of nine studies from the PDGene dataset that had not adjusted for population stratification (see Table S2).

References

1. Fung HC, Scholz S, Matarin M, Simon-Sanchez J, Hernandez D, et al. (2006) Genome-wide genotyping in Parkinson's disease and neurologically normal controls: First stage analysis and public release of data. Lancet Neurology 5: 911-916.

2. Simon-Sanchez J, Schulte C, Bras JM, Sharma M, Gibbs JR, et al. (2009) Genome-wide association study reveals genetic risk underlying Parkinson's disease. Nature Genetics 41: 1308-1312.

3. Pankratz N, Wilk JB, Latourelle JC, DeStefano AL, Halter C, et al. (2009) Genomewide association study for susceptibility genes contributing to familial Parkinson disease. Human Genetics 124: 593-605.

4. Edwards TL, Scott WK, Almonte C, Burt A, Powell EH, et al. (2010) Genome-wide association study confirms SNPs in SNCA and the MAPT region as common risk factors for Parkinson disease. Annals of Human Genetics 74: 97-109.

5. Hamza TH, Zabetian CP, Tenesa A, Laederach A, Montimurro J, et al. (2010) Common genetic variation in the HLA region is associated with late-onset sporadic Parkinson's disease. Nature Genetics 42: 781-785.

6. Pankratz N, Beecham GW, DeStefano AL, Dawson TM, Doheny KF, et al. (2012) Meta-analysis of Parkinson's disease: Identification of a novel locus, RIT2. Annals of Neurology 71: 370-384.

7. Hughes AJ, Daniel SE, Kilford L, Lees AJ. (1992) Accuracy of clinical diagnosis of idiopathic parkinson's disease: A clinico-pathological study of 100 cases. Journal of Neurology, Neurosurgery, and Psychiatry 55: 181-184.

8. Scott WK, Nance MA, Watts RL, Hubble JP, Koller WC, et al. (2001) Complete genomic screen in Parkinson disease: Evidence for multiple genes. JAMA: The Journal of the American Medical Association 286: 2239-2244.

9. Do CB, Tung JY, Dorfman E, Kiefer AK, Drabant EM, et al. (2011) Web-based genome-wide association study identifies two novel loci and a substantial genetic component for Parkinson's disease. PLoS Genetics 7: e1002141.

10. International Parkinson Disease Genomics Consortium, Nalls MA, Plagnol V, Hernandez DG, Sharma M, et al. (2011) Imputation of sequence variants for identification of genetic risks for Parkinson's disease: A meta-analysis of genome-wide association studies. Lancet 377: 641-649.

11. International Parkinson's Disease Genomics Consortium (IPDGC), Wellcome Trust Case Control Consortium 2 (WTCCC2). (2011) A two-stage meta-analysis identifies several new loci for Parkinson's disease. PLoS Genetics 7: e1002142.

12. Greco V, De Marco EV, Rocca FE, Annesi F, Civitelli D, et al. (2011) Association study between four polymorphisms in the HFE, TF and TFR genes and Parkinson's disease in southern Italy. Neurological Sciences : Official Journal of the Italian Neurological Society and of the Italian Society of Clinical Neurophysiology 32: 525-527.

13. Halling J, Petersen MS, Grandjean P, Weihe P, Brosen K. (2008) Genetic predisposition to parkinson's disease: CYP2D6 and HFE in the Faroe Islands. Pharmacogenetics and Genomics 18: 209-212.

14. Guerreiro RJ, Bras JM, Santana I, Januario C, Santiago B, et al. (2006) Association of HFE common mutations with Parkinson's disease, Alzheimer's disease and mild cognitive impairment in a portuguese cohort. BMC Neurology 6: 24.

15. Dekker MC, Giesbergen PC, Njajou OT, van Swieten JC, Hofman A, et al. (2003) Mutations in the hemochromatosis gene (HFE), Parkinson's disease and parkinsonism. Neuroscience Letters 348: 117-119.

16. Borie C, Gasparini F, Verpillat P, Bonnet AM, Agid Y, et al. (2002) Association study between iron-related genes polymorphisms and Parkinson's disease. Journal of Neurology 249: 801-804.

17. Aamodt AH, Stovner LJ, Thorstensen K, Lydersen S, White LR, et al. (2007) Prevalence of haemochromatosis gene mutations in Parkinson's disease. Journal of Neurology, Neurosurgery, and Psychiatry 78: 315-317.

18. Biasiotto G, Goldwurm S, Finazzi D, Tunesi S, Zecchinelli A, et al. (2008) HFE gene mutations in a population of Italian Parkinson's disease patients. Parkinsonism & Related Disorders 14: 426-430.

19. Buchanan DD, Silburn PA, Chalk JB, Le Couteur DG, Mellick GD. (2002) The Cys282Tyr polymorphism in the HFE gene in Australian Parkinson's disease patients. Neuroscience Letters 327: 91-94.

20. Akbas N, Hochstrasser H, Deplazes J, Tomiuk J, Bauer P, et al. (2006) Screening for mutations of the HFE gene in Parkinson's disease patients with hyperechogenicity of the substantia nigra. Neuroscience Letters 407: 16-19.
